# Heat Stress Reduces Metabolic Rate While Increasing Respiratory Exchange Ratio in Growing Pigs

**DOI:** 10.3390/ani11010215

**Published:** 2021-01-17

**Authors:** Dane W. Fausnacht, Kellie A. Kroscher, Ryan P. McMillan, Luciane S. Martello, Lance H. Baumgard, Joshua T. Selsby, Matthew W. Hulver, Robert P. Rhoads

**Affiliations:** 1 Department of Animal and Poultry Sciences, Virginia Polytechnic Institute and State University, Blacksburg, VA 24061, USA; daner@vt.edu (D.W.F.); kroscher@vt.edu (K.A.K.); 2 Virginia Tech Metabolic Phenotyping Core, Virginia Polytechnic Institute and State University, Blacksburg, VA 24061, USA; mcmillr@vt.edu (R.P.M.); hulvermw@vt.edu (M.W.H.); 3 Department of Human Nutrition, Foods, and Exercise, Virginia Polytechnic Institute and State University, Blacksburg, VA 24061, USA; 4 Department of Biosystems Engineering, University of São Paulo, Pirassununga 13635-900, SP, Brazil; martello@usp.br; 5 Department of Animal Science, Iowa State University, Ames, IA 50011, USA; baumgard@iastate.edu (L.H.B.); jselsby@iastate.edu (J.T.S.)

**Keywords:** heat stress, lipid oxidation, energy expenditure, respiratory exchange ratio

## Abstract

**Simple Summary:**

Cellular growth, particularly muscle hypertrophy, requires substantial energetics. In animals, proper substrate utilization is essential. Maximal growth is dependent on both carbohydrates and lipids being oxidized to provide as much energy as possible for protein syntheses, while amino acids must be spared from oxidation to provide an ample supply of proteogenic building blocks. However, maximal cellular growth is not a default metabolic state and is easily overridden by environmental factors such as heat stress (HS). HS has previously been hypothesized to increase metabolic rate, as is typical of a general stress response. Newer evidence, however, suggest that HS may limit energy production by inhibiting the use of lipids as a fuel source. HS metabolism instead depends on carbohydrates and even amino acids as energetic substrate, potentially limiting energy production. This study demonstrates that, contrary to current recommendations, HS reduces metabolic rate. Findings demonstrated a 38% reduction in relative energy expenditure (kcal/day/kg) due to HS. HS also caused a 33% increase in amino acid oxidation. A combination of decreased energy production and increased amino acid oxidation creates a metabolic state with severely limited growth potential which cannot be solved by simply increasing feed.

**Abstract:**

Heat stress (HS) diminishes animal production, reducing muscle growth and increasing adiposity, especially in swine. Excess heat creates a metabolic phenotype with limited lipid oxidation that relies on aerobic and anaerobic glycolysis as a predominant means of energy production, potentially reducing metabolic rate. To evaluate the effects of HS on substrate utilization and energy expenditure, crossbred barrows (15.2 ± 2.4 kg) were acclimatized for 5 days (22 °C), then treated with 5 days of TN (thermal neutral, 22 °C, *n* = 8) or HS (35 °C, *n* = 8). Pigs were fed ad libitum and monitored for respiratory rate (RR) and rectal temperature. Daily energy expenditure (DEE) and respiratory exchange ratio (RER, CO2:O2) were evaluated fasted in an enclosed chamber through indirect calorimetry. Muscle biopsies were obtained from the longissimus dorsi pre/post. HS increased temperature (39.2 ± 0.1 vs. 39.6 ± 0.1 °C, *p* < 0.01) and RER (0.91 ± 0.02 vs. 1.02 ± 0.02 VCO2:VO2, *p* < 0.01), but decreased DEE/BW (68.8 ± 1.7 vs. 49.7 ± 4.8 kcal/day/kg, *p* < 0.01) relative to TN. Weight gain (*p* = 0.80) and feed intake (*p* = 0.84) did not differ between HS and TN groups. HS decreased muscle metabolic flexibility (~33%, *p* = 0.01), but increased leucine oxidation (~35%, *p* = 0.02) compared to baseline values. These data demonstrate that HS disrupts substrate regulation and energy expenditure in growing pigs.

## 1. Introduction

Heat stress (HS) presents a complex and comprehensive challenge to an organism’s metabolism. Almost all tissue types undergo significant alterations in substrate metabolism in response to HS (brain [1], muscle [2], liver [2], intestine [3], etc.). There are a variety of hormonal adaptations to HS, particularly those associated with a general stress response (i.e., cortisol, nor/epinephrine, thyroid hormone, etc. [4]). However, increases in temperature can directly influence substrate preference in muscle tissue independent of hormonal changes. Unlike typical stress responses that are associated with increased energy expenditure and decreased respiratory quotient [5,6], HS acting independent of a hormonal response has the potential to dramatically limit energy production through reductions in lipolysis and lipid oxidation [2,3,4].

As with neuronal [7] and immune cells [8], muscle fibers express heat-sensing TRPV1 receptors. However, muscle TRPV1 receptors are housed internally on the sarcolemma and, rather than peripherally signaling the sensation of heat, locally coordinate substrate selection through the activation of heat shock factors (HSFs) and subsequent expression of heat shock proteins (HSPs). As a whole, the cellular response to heat suppresses lipolysis [9] and prioritizes lipid storage [10] while increasing cellular glucose uptake [11,12] and glycolysis [13]. The net result of these changes is a metabolic phenotype with limited lipid oxidation that relies on aerobic and anaerobic glycolysis as the predominant source of energy production. Though changes in HSP expression are not typically detected in in vivo HS models, the metabolic effects persist [2,14].

Efficient lean tissue accretion is reliant on an animal devoting a large portion of energy towards developing lean mass [15,16] however, excess accumulated heat promotes a shift in metabolic substrate preference, limiting energy production by blunting lipid oxidation and increasing lipogenesis [2]. If energy production is limited, then hypertrophy will be blunted [15], and substrates will instead be diverted towards storage as is observed in HS animals [17]. The slowed growth and reduced intake during HS have led to the assumption that animals must be in a caloric deficit [18,19]. However, due to HS-induced mechanistic adaptations to metabolism, excess caloric intake may exacerbate metabolic dysfunction. By limiting fat oxidation, even in a caloric surplus, HS could still diminish lean tissue growth by instead diverting fat towards storage, as is observed in metabolic syndrome [20,21].

While the impact of HS on numerous tissues is emerging, the effects of HS on total energy expenditure and basal metabolic rate are largely unknown. During HS, endogenous heat production becomes detrimental and priorities are shifted to promote lowering of core temperature [22,23] via evaporative cooling and behavioral adaptations including food avoidance and decreased physical activity [24]. Therefore, the purpose of this study was (1) to determine to what extent HS can influence shifts in total body substrate oxidation (RER) and (2) to determine if changes in substrate oxidation (RER) coincide with reduced overall metabolic rate.

## 2. Materials and Methods

### 2.1. Experimental Design

All procedures involving animals were approved by the Virginia Tech Institutional Animal Care and Use Committee (IACUC protocol number 19-114). Sixteen crossbred barrows were selected based on weight (range: 12–20 kg, mean 15.2 kg), individually housed in metabolic crates and fed commercial feed ab libitum throughout the entire experiment. Pigs were acclimated under thermoneutral conditions (14 h light/10 h dark, 21.6 ± 0.8 °C, with humidity 46–64%) for five days prior to treatment. Pigs were then randomly assigned (*n* = 8 per group) to thermal neutral (TN, 22.0 ± 0.4 °C, with humidity 43–63%) or heat stress (HS, 33.6 ± 0.5 °C, with humidity 22–40%) treatment groups for five days. Feed intake is reported as a daily average during the acclimation period and during the treatment period. Access to feed was temporarily restricted at 0000 h prior to indirect calorimetry and muscle biopsies and resumed immediately following these procedures. Rectal temperatures and respiratory rates were taken twice daily (0800, 2000 h) during acclimation and thrice daily (0800, 1400, 2000 h) during treatment. Rectal temperatures are reported as an average of the acclimation period (pre) and the treatment period (post).

### 2.2. Indirect Calorimetry

Pigs were placed in a controlled air plexiglass cage with a rubberized floor mat. Dimensions of the cage allowed 0.5 m^2^ of floor space for the animals to move if desired and measured approximately 1.0 m in depth by 0.5 m in width by 0.5 m in height for a volume of 0.25 m^3^. Pigs were evaluated after an 8-h overnight fast two days prior to the start of treatment and on the fifth day of treatment. Indirect calorimetry was performed between 0800 and 1200 h in treatment rooms under TN or HS conditions. Animals remained awake and were permitted to move. After a 60-min habituation period in the chamber, oxygen consumption and carbon dioxide production were measured each minute for 60 min using a ventilated chamber system (Delattre Metabolic Monitor, Sensor Medics Corp., Yorba Linda, CA, USA). Respiratory exchange ratio (RER, CO_2expired_:O_2consumed_) and daily energy expenditure (DEE) were calculated over the 60 min assessment using the Weir formula [25]. Values are reported as DEE rather than resting energy expenditure (RER), as the animals were not restricted from movement and allowed enrichment (rubberized bone) per institutional IACUC requirements.

### 2.3. Skeletal Muscle Metabolic Measures

Prior to the start of environmental treatments, muscle biopsies were taken from pigs while anesthetized under isoflurane after an overnight fast as previously described [2]. Biopsy sites were shaved and sterilized. Muscle tissue was removed from the longissimus dorsi through an incision made at about the first lumbar vertebra. A 10-gauge × 9 cm long Vacora Bard Biopsy Instrument (Bard, Murray Hill, NJ, USA) was used to harvest tissue from the biopsy sites. Approximately 90 mg of skeletal muscle tissue was collected and placed in SET buffer (0.25 M sucrose, 1 mM EDTA, 0.01 M Tris·HCl, and 2 mM ATP) for metabolic measures. After environmental treatment, biopsies were taken from the same approximate location on the contralateral side.

For fatty acid oxidation (FAO) (palmitate ([1–14C] palmitic acid) and pyruvate oxidation ([1–14C] pyruvate)) and metabolic flexibility measures, muscle tissue was homogenized as previously described [2,26]. Palmitate oxidation (FAO-CO_2_) was evaluated through 14CO_2_ production and 14C-labeled acid-soluble metabolites (FAO-ASM) from the oxidation of [1–14C] palmitic acid, the combination of palmitate oxidation and acid-soluble metabolites was referred to as the total fat uptake (total-FAO). Pyruvate oxidation was assessed by measuring 14CO_2_ production from the oxidation of [1–14C] pyruvate. Metabolic flexibility was evaluated by comparing [1–14C] pyruvate oxidation in the presence and absence of 100 μM palmitic acid. The ability of free fatty acid (FFA) to suppress glucose oxidation was used as a representative of metabolic flexibility with a greater reduction in pyruvate oxidation in the presence of palmitate indicating greater metabolic flexibility [26].

### 2.4. Statistical Analysis

Statistical analyses were performed using PRISM software (Prism version 8.0.0 for Windows, GraphPad Software, La Jolla, CA, USA). Correlations were performed using simple liner regression with an F-test used to determine statistical significance. Pre/post treatment measures (weight, RER, DEE, and metabolic measures) vs. treatment (TN/HS) were analyzed through repeated measures two-way ANOVA. Periodical measurements (feed intake, rectal temperatures, and respiratory rates) were analyzed using a restricted maximum likelihood (REML) mixed-effect analysis with repeated measures, where values are reported as the average within acclimation or treatment periods. Tukey and Sidak’s post hoc analyses determined environmental effects within timepoints and time effects within treatments, respectively. Pig variance was included as a random variable. The model included the treatment (TN and HS) and treatment-by-time point interaction. Data are reported as mean ± SEM and were considered significant at *p* < 0.05 and defined as a trend if >0.05 but <0.10.

## 3. Results

### 3.1. Growth and Thermoregulatory Response

**Figure 1:**
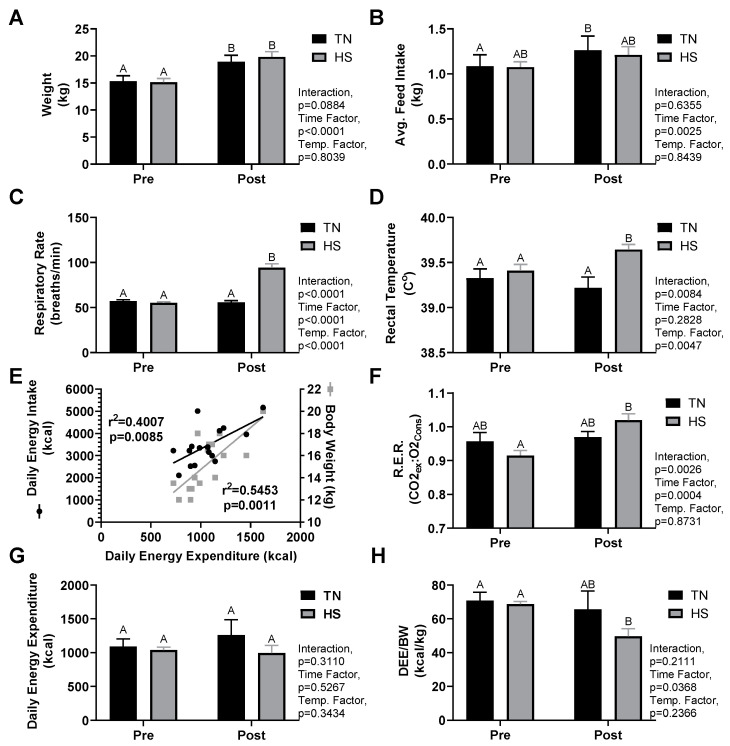
Effect of environmental conditions on growing pig weight (**A**), feed intake (**B**), respiratory rate (**C**), rectal temperature (**D**). Correlation of daily energy expenditure (DEE) to animal body weight and energy intake (**E**). Indirect calorimetry measures on growing pigs of respiratory exchange ratio (RER) (**F**), DEE (**G**), and DEE per body weight (BW) (**H**). Pigs were acclimated under thermoneutral conditions (14 h light/10 h dark, 21.6 ± 0.8 °C, with humidity 46–64%) for five days prior to treatment (Pre). Pigs were then randomly assigned (*n* = 8 per group) to thermal neutral (TN, 22.0 ± 0.4 °C, with humidity 43–63%) or heat stress (HS, 33.6 ± 0.5 °C, with humidity 22–40%) treatment groups for five days (Post). Means without a shared letter are significantly different, *p* < 0.05.

Growth and thermoregulatory parameters are summarized in Table 1 and Figure 1A–D. Pigs in both the TN and HS groups experienced similar weight gain during the study (*p* < 0.01 time; *p* = 0.80 treatment). There was no difference in feed intake prior to treatment (*p* > 0.99) nor was there an effect of treatment on feed intake (*p* = 0.84). A time effect was detected for feed intake (*p* < 0.01) with further analysis revealing the TN group did experience a significant increase in feed intake (*p* = 0.02) form pre to post while the HS group only trended on a change (*p* = 0.07). Respiratory rates (BPM) increased due to HS treatment (55 ± 1 vs. 94 ± 4, *p* < 0.01) and were higher than TN controls (*p* < 0.01). Rectal temperatures of HS animals increased due to treatment (pre 39.4 ± 0.1 °C vs. post 39.6 ± 0.1 °C, *p* = 0.02) and were higher than those of TN pigs post treatment (39.2 ± 0.1 °C, *p* = 0.02).

### 3.2. Indirect Calorimetry

Indirect calorimetry parameters are summarized in Figure 1E–H. For both groups, pretreatment measures of DEE correlated with pig body weight (r^2^ = 0.55, *p* < 0.01) and average daily feed intake (r^2^ = 0.40, *p* < 0.01). RER was unchanged in TN animals (*p* = 0.74) but increased in the HS group (pre-HS 0.91 ± 0.02 vs. post-HS 1.02 ± 0.02, *p* < 0.01). DEE did not differ between TN (pre 1090 ± 120 kcal vs. post 1252 ± 238 kcal) and HS (pre 1038 ± 48 kcal vs. post 992 ± 123 kcal) groups prior to treatment nor was there an effect of treatment (*p* = 0.34). Relative energy expenditure did not exhibit a group or interaction effect, but a significant time effect was detected from pre to post treatment (*p* = 0.04). Though both TN and HS groups experienced a decrease in relative energy expenditure, a Sidak’s post hoc analysis revealed that this drop was only significant in the HS group (pre-HS 68.8 ± 1.7 kcal/kg vs. post-HS 49.7 ± 4.8 kcal/kg, *p* < 0.01).

### 3.3. Substrate Metabolism

**Figure 2:**
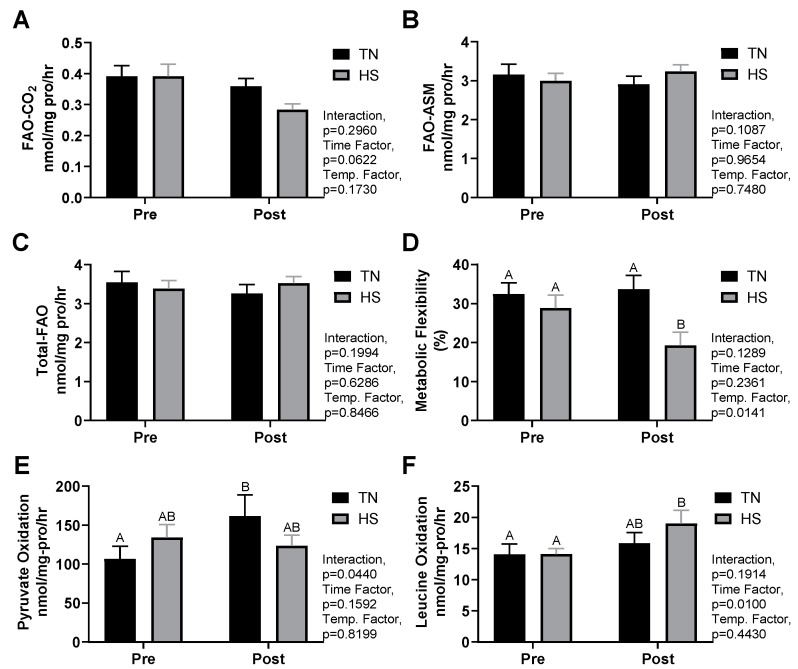
Effect of environmental conditions on growing pigs for measures of complete fatty acid oxidation (FAO-CO_2_) (**A**), acid soluble metabolites (incomplete oxidation) (FAO-ASM) (**B**), total-FAO (FAO-CO_2_ + FAO-ASM) (**C**), metabolic flexibility (pyruvate oxidation:pyruvate oxidation +100 µM palmitic acid) (**D**), pyruvate oxidation (**E**), and leucine oxidation (**F**). Pigs were acclimated under thermoneutral conditions (14 h light/10 h dark, 21.6 ± 0.8 °C, with humidity 46–64%) for five days prior to treatment (Pre). Pigs were then randomly assigned (*n* = 8 per group) to thermal neutral (TN, 22.0 ± 0.4 °C, with humidity 43–63%) or heat stress (HS, 33.6 ± 0.5 °C, with humidity 22–40%) treatment groups for five days (Post). Means without a shared letter are significantly different, *p* < 0.05.

Muscle oxidative measures are summarized in Figure 2. There were no differences between groups for any of the muscle homogenate measures prior to treatment. Measures of FAO (FAO-CO_2_, FAO-ASM, and total-FAO) did not differ due to treatment, although complete FAO-CO_2_ did tend to drop in the HS group (*p* = 0.06) (Figure 2A–C). Metabolic flexibility was reduced in the HS group post-treatment compared to TN (19.2 ± 3.7% vs. 33.7 ± 3.8%, *p* < 0.01, Figure 2D). Pyruvate oxidation rates increased in the TN group after the treatment period (62%, *p* = 0.04), though no change was observed in HS pigs (Figure 2E). A time effect was observed for leucine oxidation rates (*p* = 0.01). A further Sidak’s analysis demonstrated that leucine oxidation was elevated after HS treatment (38 ± 18%, *p* = 0.02), but not significantly affected by TN (*p* = 0.48) (Figure 2F). Post metabolic flexibility measures were correlated to change in RER (r^2^ = 0.40, *p* < 0.01) and RR (r^2^ = 0.36, *p* = 0.01), and tended to be correlated with change in rectal temperature (r^2^ = 0.24, *p* = 0.06). Pyruvate oxidation was negatively correlated to changes in rectal temperature (r^2^ = 0.28, *p* = 0.04) (graphs not shown).

### 3.4. HS Metabolism and Performance

**Figure 3:**
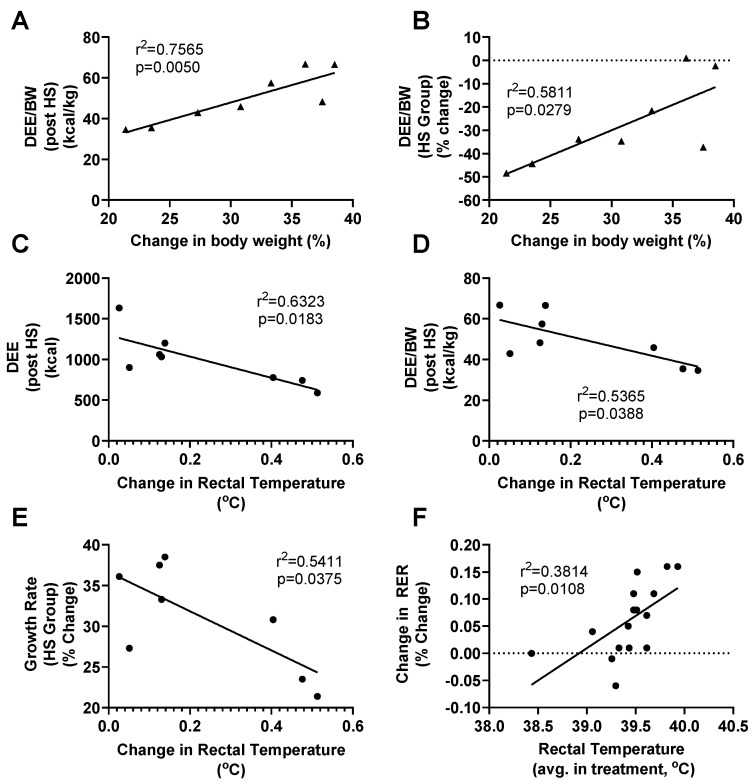
Relationship of daily energy expenditure (DEE) per bodyweight (BW) (post treatment, (**A**), and effect of treatment (**B**) compared to animal growth rates after 5 days of heat stress (HS)). Relationship of daily energy expenditure (**C**), daily energy expenditure per bodyweight (**D**) and growth rate (**E**) compared to change in rectal temperature during days after HS. Changes in RER after 5 days of HS or thermal neutral environmental treatments compared to average rectal temperature during treatment (**F**). Pigs were acclimated under thermoneutral conditions (14 h light/10 h dark, 21.6 ± 0.8 °C, with humidity 46–64%) for five days prior to treatment. Pigs were then randomly assigned (*n* = 8 per group) to thermal neutral (TN, 22.0 ± 0.4 °C, with humidity 43–63%) or heat stress (HS, 33.6 ± 0.5 °C, with humidity 22–40%) treatment groups for five days.

Relative energy expenditure post HS was correlated to growth (r^2^ = 0.77, *p* < 0.01, Figure 3A). Reductions in energy expenditure per body weight due to HS were also correlated to growth (r^2^ = 0.58, *p* = 0.03, Figure 3B), with the pigs who experienced the greatest decrease in energy expenditure per body weight also experiencing the smallest growth. DEE (r^2^ = 0.63, *p* = 0.02, Figure 3C) and DEE/BW (r^2^ = 0.54, *p* = 0.04, Figure 3D) of HS animals were negatively correlated with change in rectal temperature where pigs with the greatest increases in rectal temperature due to HS presented with lower rates of exergy expenditure post HS. Changes in rectal temperature were also negatively correlated with growth rates (r^2^ = 0.54, *p* = 0.04, Figure 3E), such that pigs with the greatest increases in core temperature exhibited the slowest rates of growth. A positive relationship between change in RER and change in rectal temperature is seen when both TN and HS groups were analyzed together (r^2^ = 0.38, *p* = 0.01, Figure 3F).

## 4. Discussion

The major findings of this study are that (1) HS caused an increase in RER and (2) HS decreased metabolic rate. These findings are in opposition of typical stress responses which show increased metabolic rate and FAO [5,6]. This paper is the first to demonstrate a reduction in relative energy expenditure due to HS. Increases in RER are consistent with findings from both acute and chronic HS studies, which highlight increased reliance on glucose as a fuel substrate [12,27,28] paired with suppressed lipid oxidation [2,14]. The extent to which HS increased RER in this study is, however, notable, as fasting values above 1.00 indicate minimal contributions of lipid oxidation to metabolism and either anaerobic energy production or de novo lipogenesis [29,30].

The differences in relative energy expenditure between the TN and HS groups post treatment driven by an increased DEE in TN that was not observed in HS. The increase in TN DEE (~17%) can be attributed to the weight gain (~24%) of TN animals, as weight is highly correlated to energy expenditure [31]. Lower oxidative capacity overall may explain the differences in DEE, as the TN group observed an increase in pyruvate oxidation that was not detected in HS, and HS trended on a reduction in complete FAO that was not detected in the TN group. It should be noted that while feed intake was not different between TN and HS during acclimation or treatment, TN animals did exhibit a statistically significant increase in feed intake from pre to post (*p* = 0.02) while the HS group trended towards an increase (*p* = 0.07).

While the effects of HS on metabolic rate have not been extensively evaluated, NRC recommendations [32] state that HS increases energy demand and feed energy should be increased to compensate. These recommendations are based on acute studies of environmental temperature on metabolic rate which indicate that temperature stress (hot or cold), creates a J-shaped response in metabolic rate [33,34,35,36], such that metabolic rates increase proportionally with environmental extremes. The degree of HS for this study was relatively mild and reductions in weight gain and feed intake were not observed, yet the reductions in energy expenditure persisted. This would indicate the pigs were in a positive energy balance with excess energy most likely stored as fat. Following current recommendations [32] of increasing energy intake during HS could result in overfeeding, which would exacerbate the metabolic disruptions associated with HS. Diet-induced obesity (DIO)/diabetes and HS share many parallels in their mechanistic disruption of substrate metabolism. Both DIO/diabetes and HS persist with impaired lipid metabolism, decreased metabolic flexibility, increased reliance on carbohydrates as a fuel source, and ultimately increased adiposity [2,14,37].

The data herein support a new association between HS and decreased energy expenditure highlighting a potential role for HS as a causal factor in the development of obesity, as increased core temperatures have been reported in obese populations [38,39]. That is, obesity drives metabolic dysregulation, this dysregulation may be exacerbated or complicated by increased core temperatures, which also appears to drive independent metabolic dysregulation. This may be particularly relevant to obese populations living in hot environments like southern United States or in areas that trap heat such as urban centers [40]. These data also raise the possibility that treatments designed to ameliorate the metabolic effects of obesity may also be viable as treatments for HS.

RER is a whole-body measure of substrate metabolism. However, due to the lean composition of the production pig and the large contribution of skeletal muscle to overall body mass, skeletal muscle metabolism has major influence over RER values in this species. This study as well as our previous reports [2,41] have demonstrated that HS causes decreases in in situ muscle metabolic flexibility. As measured, a reduction in metabolic flexibility is an indication that pyruvate oxidation is the preferred means of energy production in muscle tissue regardless of lipid availability. Consistent with this, the current study demonstrates a rise in RER due to HS, where post-HS values of 1.02 ± 0.02 VCO_2_/VO_2_ indicate that carbohydrates are the primary substrate of oxidation while capacity for FAO is limited. Reduced capacity for FAO can limit muscle hypertrophy [15,42] and can lead to fat accumulation [43] and even myosteatosis [17]. RER values above 1.00 (in a sedentary state) are suggestive of de novo lipogenesis [29,30]. These findings indicate that HS pigs rarely catabolize lipid stores and that nearly all substrate used for energy production is coming directly from dietary intake of carbohydrates or liver gluconeogenesis possibly fueled by increased proteolysis [29,44,45].

Increased proteolysis also appears to provide amino acids (AAs) as alternate and direct fuel sources to muscle tissue, as leucine oxidation increased 35% following HS. Oxidation of branch chain amino acids typically only becomes a significant energy source during prolonged exercise or extended fasting, generally stimulated by rising cortisol levels and elevated proteolysis [46]. Other studies have reported increased proteolysis due to HS [47,48], which was previously thought to provide substrate for gluconeogenesis [45,49]. However, data from this study suggest muscle tissue may mobilize and oxidize significant levels of AAs during HS along with increased glucose oxidation, possibly to compensate for the blunted lipid metabolism. It should be noted that measures from this study looked only at leucine oxidation and it is not known how the oxidation rates of other amino acids are influenced by HS.

## 5. Conclusions

Data from the current study indicate HS suppresses metabolic rate mostly due to HS-induced suppression of lipid oxidation as indicated by elevated RER levels and reduced muscle fatty acid oxidation. Heat stress also caused increased leucine oxidation in muscle, possibly as a mechanism to compensate for the loss of lipid oxidation. The net effect of HS is a metabolic phenotype that closely resembles overfeeding, possibly leading to an adiposity phenotype shared by HS and overfeeding conditions.

## Figures and Tables

**Table 1:** Summary of growth, thermoregulatory, and indirect calorimetry measures.

Measure	Thermal Neutral		Heat Stress		Interaction	Time Effect	Temperature Effect
	Pre	Post	Pre	Post			
Weight (kg)	15.3 ± 1.1	18.9 ± 1.3 *	15.1 ± 0.7	19.8 ± 1.0 *	0.0884	<0.0001	0.8039
Rectal Temp. (°C)	39.3 ± 0.1	39.2 ± 0.1	39.4 ± 0.1	39.6 ± 0.1 *^t^	0.0084	0.2828	0.0470
Respiratory Rate (B.P.M.)	57 ± 2	56 ± 2	55 ± 1	94 ± 4 *^t^	<0.0001	<0.0001	<0.0001
Feed Intake (kg/day)	1.1 ± 0.1	1.3 ± 0.2 *	1.1 ± 0.1	1.2 ± 0.1	0.6355	0.0025	0.8439
RER (CO_2expired_:O_2consumed_)	0.95 ± 0.03	0.97 ± 0.01	0.91 ± 0.02	1.02 ± 0.02 *	0.0026	0.0004	0.8731
DEE (kcal)	1090 ± 120	1252 ± 238	1038 ± 48	992 ± 123	0.3110	0.5267	0.3434
DEE/BW (kcal/kg)	70.9 ± 5.1	67.5 ± 11.6	68.8 ± 1.7	49.7 ± 4.8 *	0.2111	0.0368	0.2366

Pigs were acclimated under thermoneutral conditions (14 h light/10 h dark, 21.6 ± 0.8 °C, with humidity 46–64%) for five days prior to treatment (Pre). Pigs were then randomly assigned (*n* = 8 per group) to thermal neutral (TN, 22.0 ± 0.4 °C, with humidity 43–63%) or heat stress (HS, 33.6 ± 0.5 °C, with humidity 22–40%) treatment groups for five days (Post). DEE (daily energy expenditure), RER (respiratory exchange ratio). *p*-values were generated with two-way repeated measures ANOVA. (*) indicates time effect within treatment (*p* < 0.05). (t) indicates a temperature effect within time point (*p* < 0.05).

## Data Availability

The data presented in this study are available on request from the corresponding author.
